# 

**Published:** 1895-05-11

**Authors:** 


### CONTENTS.

The Royal Medical Benevolent College . . . 89

The Scourge of India . . . . . . . . . 90

Modern Medico-Psychology and Psychiatry. By P. S. Clouston, M.D. . . . . . . . . 91

Modern Sociology.—Poverty and Pensions . . . . . 92

Annotations.—A Bombay M.B.—Then and Now in the Village—Oxford and its Infirmary . . . . . . . . 93

Notes and News . . . . . . . . . . 94

Scraps and Gleanings . . . . . . . . . 106

The Student of Medicine . . . . . . . . 106

Medical Progress and Hospital Clinics—
Intestinal Anastomosis . . . . . . . . 95
Progress in Medicine.—Kidney Diseases . . . . . . 97
Diabetes . . . . . . . . . . 98
Progress in Surgery.—Surgery of the Vermiform Appendix . 99
New Appliances and Things Medical . . . . . 100
Editor's Letter-Box . . . . . . . . . . 100
The Institutional Workshop:
Hospital Administration.—Visitors to Hospitals and Asylums 101
Hospital Festivals and Meetings . . . . . . . 102

### EXTRA SUPPLEMENT.—THE NURSING MIRROR.

News from the Nursing World.—Our Princess—Fire at a Fever Hospital—Wards and Washing—St. Luke's House—A Cottage Hospital—The Guardians of the Poor—Queen's Nurse at Wellingborough—District Nurses in Somerset—Nurses' Earnings—Lewes—A Fête at Derby—Quarantine at Melbourne—Nursing in Victoria—Stockton and Thornaby—Nursing at Hyderabad—Short Items . . . . . xxxix

Dress and Uniforms.—The Middlesex Hospital . . . . xlii

Elementary Anatomy and Surgery for Nurses. By W. McAdam Eccles, M.B., M.S., F.R.C.S. . . . . . . xli
Death in Our Ranks—Appointments . . . . . . xlii
Everybody's Opinion . . . . . . . . . xliii
Novelties for Nurses . . . . . . . . . xliii
Hospital Secretaries at a Discount . . . . . . xliii
A Nurses Pension Fund for Sweden . . . . . . xliii
Notes and Queries . . . . . . . . . . xliv

